# Supplementary material for: Contextualising person-centred mental health and psychosocial support (MHPSS) services: A qualitative study of the preferences and experiences of displaced Syrians in Northwest Syria and Türkiye
Source: Glob Ment Health (Camb). 2025 Sep 8;12:e102. doi: 10.1017/gmh.2025.10058 (PMC12455514; doi:10.1017/gmh.2025.10058)
Supplement: McGrath et al. supplementary material [file S2054425125100587sup001.docx]

**SUPPLEMENTARY MATERIAL**

**Survey instrument**

| **Item** | **English** | **Arabic** |
| --- | --- | --- |
| CSQ 1 | How would you rate the quality of service you received? | كيف تقيم جودة الخدمة التي تلقيتها؟ |
| CSQ 2 | Did you get the kind of service you wanted? | هل حصلت على نوع الخدمات التي رغبت بها؟ |
| CSQ 2a | - What kind of help were you hoping for? | ما نوع المساعدة التي كنت تتمناها؟ |
| CSQ 2b | - What kind of help did you get? | ما نوع المساعدة التي حصلت عليها؟ |
| CSQ 3 | To what extent has your interaction with *[practitioner name]* met your needs? | إلى أي مدى لبت الخدمات التي تلقيتها من *[اسم الأخصائي]* احتياجاتك؟ |
| CSQ 3a | - What needs were met? | ما الاحتياجات التي تمت تلبيتها؟ |
| CSQ 3b | - What needs were not met? | ما الاحتياجات التي لم يتم تلبيتها؟ |
| CSQ 4 | If a friend needed similar help, would you recommend the service to them? | إذا كان صديقك بحاجة لنفس الخدمة بتنصحو يتلقاها من هالمركز؟ |
| CSQ 4a | - Could you tell me why? | هل تستطيع إخباري لماذا؟ |
| CSQ 5 | How satisfied are you with the amount of help you received? | هل أنت راضٍ عن حجم المساعدة التي تلقيتها؟ |
| CSQ 6 | Have the services you received helped you to deal more effectively with your problems? | هل ساعدتك الخدمات التي تلقيتها في التعامل بكفاءة أكثر مع مشاكلك؟ |
| CSQ 6a | - Could you tell me how? | هل يمكنك ان تخبرني كيف؟ |
| CSQ 7 | In an overall, general sense, how satisfied are you with the service you received? | بشكل عام، إلى أي درجة أنت راضِ عن الخدمات اللي حصلت عليها؟ |
| CSQ 8 | If you were to seek help again, would you come back to our service? | إذا كنت تبحث عن مساعدة مرة أخرى فهل ستعاود استخدام خدماتنا؟ |
| CSQ 8a | - Could you tell me why? | هل تستطيع أن تخبرني لماذا؟ |
|  |  |  |
| A1 | What was the most useful idea that you received from your MHPSS worker? | خلال الجلسة التي تلقيتها، ما هي الفكرة المفيدة جداً التي حصلت عليها من موظف الصحة النفسية والدعم النفسي الاجتماعي؟ |
| A2 | How will you use this idea in your future life? | كيف ستستخدم هذه الفكرة في حياتك المستقبلية؟ |
